# Opposing Effects of Particle Pollution, Ozone, and Ambient Temperature on Arterial Blood Pressure

**DOI:** 10.1289/ehp.1103647

**Published:** 2011-10-21

**Authors:** Barbara Hoffmann, Heike Luttmann-Gibson, Allison Cohen, Antonella Zanobetti, Celine de Souza, Christopher Foley, Helen H. Suh, Brent A. Coull, Joel Schwartz, Murray Mittleman, Peter Stone, Edward Horton, Diane R. Gold

**Affiliations:** 1 Department of Environmental Health, Harvard School of Public Health, Boston, Massachusetts, USA; 2 IUF-Leibniz Research Institute for Environmental Medicine and Medical Faculty, University of Düsseldorf, Düsseldorf, Germany; 3 Joslin Diabetes Center, Boston, Massachusetts, USA; 4 National Opinion Research Center (NORC) at the University of Chicago, Chicago, Illinois, USA; 5 Department of Biostatistics, Harvard School of Public Health, Boston, Massachusetts, USA; 6 Beth Israel Deaconess Medical Center, Harvard Medical School, Boston, Massachusetts, USA; 7 Brigham and Women’s Hospital, Boston, Massachusetts, USA; 8 Channing Laboratory, Harvard Medical School, Boston, Massachusetts, USA

**Keywords:** air pollution, ambient temperature, blood pressure, diabetes mellitus, epidemiology, ozone, particles

## Abstract

Background: Diabetes increases the risk of hypertension and orthostatic hypotension and raises the risk of cardiovascular death during heat waves and high pollution episodes.

Objective: We examined whether short-term exposures to air pollution (fine particles, ozone) and heat resulted in perturbation of arterial blood pressure (BP) in persons with type 2 diabetes mellitus (T2DM).

Methods: We conducted a panel study in 70 subjects with T2DM, measuring BP by automated oscillometric sphygmomanometer and pulse wave analysis every 2 weeks on up to five occasions (355 repeated measures). Hourly central site measurements of fine particles, ozone, and meteorology were conducted. We applied linear mixed models with random participant intercepts to investigate the association of fine particles, ozone, and ambient temperature with systolic, diastolic, and mean arterial BP in a multipollutant model, controlling for season, meteorological variables, and subject characteristics.

Results: An interquartile increase in ambient fine particle mass [particulate matter (PM) with an aerodynamic diameter of ≤ 2.5 μm (PM_2.5_)] and in the traffic component black carbon in the previous 5 days (3.54 and 0.25 μg/m^3^, respectively) predicted increases of 1.4 mmHg [95% confidence interval (CI): 0.0, 2.9 mmHg] and 2.2 mmHg (95% CI: 0.4, 4.0 mmHg) in systolic BP (SBP) at the population geometric mean, respectively. In contrast, an interquartile increase in the 5-day mean of ozone (13.3 ppb) was associated with a 5.2 mmHg (95% CI: –8.6, –1.8 mmHg) decrease in SBP. Higher temperatures were associated with a marginal decrease in BP.

Conclusions: In subjects with T2DM, PM was associated with increased BP, and ozone was associated with decreased BP. These effects may be clinically important in patients with already compromised autoregulatory function.

Persons with diabetes were at higher risk of dying during recent European heat waves (Schifano et al. 2009) and had increased rates of cardiovascular death when exposed to elevated levels of particulate matter (PM) pollution (Goldberg et al. 2001; Zanobetti et al. 2009). The potential biological mechanisms that mediate this hypothesized increased susceptibility to environmental effects include heat-associated orthostatic hypotension (Crandall and González-Alonso 2010) and air-pollution–induced autonomic imbalance, oxidative stress, and systemic inflammation (Brook et al. 2010), which promote endothelial dysfunction (Madrigano et al. 2010), impaired vascular reactivity, and hypertension (Brook et al. 2009). Although it is possible that heat-associated hypotension or pollution-associated hypertension and endothelial dysfunction contribute to the risk of death, the combined effects of climate and pollution on blood pressure (BP) regulation are not well understood.

Persons with diabetes may be at greater risk for environmental perturbations of mechanisms that regulate BP. Diabetes itself increases the risk of hypertension and orthostatic hypotension because of chronic autonomic dysregulation, endothelial dysfunction, atherosclerosis, and dysregulation of fluid balance and the renin-angiotensin system (Pop-Busui 2010; Ribeiro-Oliveira et al. 2008). Common medications, such as beta blockers, may further block appropriate compensatory autoregulatory vascular responses (Bell 2009). We have shown that elevated PM pollution exposure adds to autonomic dysregulation, endothelial dysfunction (O’Neill et al. 2005), and inflammation (Dubowsky et al. 2006), particularly in persons with diabetes. Moreover, poor diabetes control increases the risk of autonomic dysfunction (Pop-Busui 2010), but it is not known whether optimal control of glucose or BP ameliorates the effects of pollution or high temperature.

In this study, we investigated the combined short-term effects of PM air pollution, ozone, and ambient temperature on systolic BP (SBP), diastolic BP (DBP), and central mean arterial BP in a panel of 70 subjects with type 2 diabetes mellitus (T2DM). We also evaluated whether BP or glucose control in persons with diabetes modulated the effects of pollution or climate.

## Methods

*Study population and design.* This analysis is based on a panel study of subjects with T2DM and was specifically designed to examine vascular and autonomic function as well as inflammatory changes associated with acute changes in air pollution and its constituents. From September 2006 through July 2010, subjects with T2DM were recruited for screening for a prospective repeated measures study if they were 40–85 years of age and lived within 25 km of the central air monitoring site in Boston. Exclusion criteria focused on

Exposures that could obscure ambient pollution measurement (e.g., secondhand tobacco smoke at home, living beyond 25 km of the central monitoring station)
Conditions with electrophysiological or vascular effects (e.g., current atrial fibrillation/flutter; history of clinically significant ventricular arrhythmias, pacemaker, or implanted defibrillator; acute myocardial infarction or stent placement within the preceding 6 months)
Clinical/biomarker parameters requiring immediate attention [e.g., uncontrolled hypertension (> 180 mmHg SBP, > 105 mmHg DBP)]
Other advanced diseases (e.g., solid organ transplant, active autoimmune disease, dementia, diabetes type 1, renal failure, seizure disorder or stroke, sleep apnea).

Five follow-up clinic visits were scheduled 2 weeks apart on the same weekday in the morning. Subjects were asked to fast for the 12 hr before the clinical measurements. Clinic visits included a personal interview on sociodemographic characteristics, health status, medical history, medication, and lifestyle; blood and urinary analysis; and clinical examinations. The study protocol was approved by the institutional review board at the Brigham and Women’s Hospital, the Joslin Diabetes Clinic, and the Harvard School of Public Health. All participants provided written informed consent.

*Air pollution and meteorology.* Ambient air pollution monitoring was conducted at a central site operated by the Harvard School of Public Health. The monitoring station was located on a rooftop approximately 500 m from the examination site. Particle measurements included PM with aerodynamic diameter ≤ 2.5 μm (PM_2.5_), sulfate (SO_4_^2–^), black carbon (BC), elemental carbon (EC), organic carbon (OC), and particle number concentration (PNC). Continuous PM_2.5_ [measured using a tapered element oscillating microbalance sampler (TEOM) 1400A; Rupprecht and Patashnick, Albany, NY, USA] represents the overall mass of particles < 2.5 μm in aerodynamic diameter. We used a season-specific correction factor, based on the data from a collocated gravimetric sampler, to compensate for semivolatile mass lost using the TEOM sampler. SO_4_^2–^ particles (measured using a sulfate particulate analyzer model 5020; Thermo Fisher Scientific, Waltham, MA, USA) are formed through secondary reactions of sulfur dioxide emitted primarily by coal-burning power plants and often transported regionally over long distances (e.g., hundreds of kilometers) (Thurston and Spengler 1985). BC (measured using a model AE-14 Aethalometer; Magee Scientific, Berkeley, CA, USA) is used to indicate traffic emissions, especially those related to diesel fuel combustion. OC (measured using an EC-OC analyzer; Sunset Laboratory Inc., Tigard, OR, USA) originates mainly from biomass and fossil fuel combustion and from secondary formation of gas-phase precursors. PNC (measured using a condensation particle counter model 3022a; TSI Inc., Shoreview, MN, USA) reflects ultrafine combustion-related particles (< 100 nm) and indexes fresh, locally generated traffic particles (Morawska and Zhang 2002).

Hourly ambient concentrations of ozone were obtained from the Massachusetts Department of Environmental Protection (Boston, MA) for the greater Boston area. We calculated hourly mean ozone concentrations from data reported by all air pollution monitoring stations. Hourly mean temperature, relative humidity, and barometric pressure measurements were collected from the National Weather Service Station at Logan Airport (East Boston) located approximately 12 km from the examination site.

We imputed missing hourly data for PM_2.5_ and BC using regression modeling, including a long-term time trend, day of week, hour of day, temperature, relative humidity, barometric pressure, and nitrogen dioxide as predictors. All collections, processing of samples, analysis, and reporting were conducted according to standard operating procedures (Dockery et al. 2005).

*BP measurements.* All clinical measurements were performed by trained study personnel in a temperature-controlled room (22–24°C). After a 5-min period of rest, brachial artery BP of the dominant arm of seated participants was measured three times at 1-min intervals, using an automated oscillometric sphygmomanometer (OSZ5; WelchAllyn, Skaneateles Falls, NY, USA). We used the average of the second and third measurement. If SBP was > 180 mmHg, the measurement was repeated after an additional 5 min of rest. If SBP remained > 180 mmHg, the study doctor was notified and came to examine and speak to the patient to determine if the study visit could continue. Central mean arterial BP was derived from pulse wave analysis, conducted with a SphygmoCor Px Pulse Wave Analysis System (model SCOR-Px; Atcor Medical Pty Ltd., Sydney, Australia). In a seated position, participants extended their dominant arm onto a flat surface, ensuring that the elbow was at heart level. Applanation tonometry of the radial artery, guided by visual inspection of the pulse pressure (PP) wave form and by a built-in quality score of the homogeneity of the observed waveforms, was conducted to record 10 sec of sequential peripheral pulse waveforms. The peripheral wave forms were transformed into corresponding central aortic waveforms via a previously validated transfer function (Chen et al. 1997). Central mean arterial BP, PP, augmentation pressure (AP), and augmentation index (AI) were derived from the aortic pressure wave. The AI is defined as the difference between the first and second systolic peak (i.e., AP), divided by PP, and expressed as a percentage: AI (%) = (AP/PP) × 100. Larger values indicate a higher pulse wave velocity and earlier return of the reflected wave, caused by increased arterial stiffness or vascular resistance. Because AP and AI are strongly dependent on heart rate, values were normalized to a heart rate of 75 beats/min (bpm).

*Covariates.* Current medications were coded according to the World Health Organization (WHO) Anatomical Therapeutic Chemical classification system at each visit (WHO Collaborating Centre for Drug Statistics Methodology 2011). Body mass index (BMI) was calculated at each visit from height (measured only at baseline) and weight measurements. Presence of coronary heart disease was assessed by review of the subjects’ medical records. Baseline lipid and HbA1c (glycosylated hemoglobin) levels were measured using standard methods. Blood glucose and C-reactive protein levels were measured at each visit using standard methods.

*Statistical analysis.* We used linear mixed models that accounted for repeated measures within one subject to estimate the effect of short-term increases in PM (PM_2.5_, SO_4_^2–^, BC, OC, PNC), ozone, and temperature on changes in SBP, DBP, and central mean arterial BP.

BP values were skewed and, therefore, were log-transformed to stabilize the variance. Mixed models included a random intercept for subject in the analysis, a first-order autoregressive term, and subject characteristics (i.e., age, sex, BMI, HbA1c), season, and ambient temperature. Exposure metrics included short-term exposure periods (mean values during the 1–5 days before the BP measurement). Estimates are given as the percent change in BP per interquartile range (IQR) change in exposure with 95% confidence intervals (CIs), or as the absolute change in BP (millimeters of mercury) calculated at the population geometric mean.

In the base models, we included one environmental exposure (PM pollution, ozone, or temperature) and controlled for season, age, sex, and BMI. Other personal characteristics (e.g., years of diabetes, HbA1c at baseline, and blood glucose level on the morning of the visit) were added if they improved model fit. Continuous variables were included as polynomials if there were signs of departure from linearity. The time course for the relation of exposure to BP was explored separately for each environmental exposure considered. We then estimated the effects of PM, ozone, and temperature in models including all of these environmental exposures, using the mean value for the time window of exposure that was most strongly (i.e., the largest point estimate) associated with BP for each exposure. In extended models, HbA1c, antihypertensive medication and blood glucose on the day of the clinic visit were included.

In sensitivity analyses, we investigated the association between PM air pollution and BP outcomes only in participants who lived within 15 km of the central measuring station. We also investigated the association by including fixed effects for subjects in the analysis to remove between-subject variation.

Using interaction terms, we evaluated baseline BP level, intake of antihypertensive medication, AI, BMI, C-reactive protein, HbA1c, duration of diabetes, and season as potential modifiers of the relation of pollution or temperature to BP. To derive interaction terms of exposure variables with continuous variables, we dichotomized the potential effect modifier at the median. Statistical analyses were performed using SAS (version 9.2; SAS Institute Inc., Cary, NC, USA).

## Results

During the study period from September 2006 through July 2010, 70 participants were enrolled in the repeated measures study, with a mean of 4.8 repeated observations each (Table 1), resulting in 322 observations with complete information on outcome and covariates. Most participants had a long-standing history of diabetes (mean, 9.9 years; median, 8.0 years). Obesity, hypertension, and antihypertensive medication were highly prevalent in this population.

**Table 1:** Baseline characteristics of 70 participants with T2DM in Boston, Massachusetts (USA): September 2006 through July 2010.

Characteristic	Mean (range) or *n* (%)	
No. of follow-up visits/subject	4.8	(1–5)
Age (years)	64.4	(45–86)
SBP (mmHg)	132	(100–179)
DBP (mmHg)	75	(58–98)
Central mean arterial pressure (mmHg)	95	(70–128)
AP at 75 bpm (mmHg)	11.6	(3.0–32.0)
AI at 75 bpm (%)	25.6	(10.0–44.3)
BMI (kg/m^2^)	31.3	(20.5–57.0)
HbA1c (%)	7.0	(5.5–10.0)
Years of diabetes*a*	9.9	(1–38)
Women	37	(53)
Race		
Caucasian	58	(83)
Black	7	(10)
Other	5	(7)
Self-reported diagnoses		
Hypertension	53	(76)
Coronary artery disease	7	(10)
Antihypertensive medication	53	(76)
Beta blockers	25	(36)
Calcium channel blockers	15	(21)
Angiotensin-converting enzyme inhibitors	30	(43)
Angiotensin II receptor blockers	19	(27)
Lipid-lowering medication	61	(87)
Platelet aggregation inhibitors	52	(74)
**a**Based on 67 observations because of missing data.		

Ambient PM_2.5_ concentrations in Boston were below the currently set U.S. daily standard during the study period (Table 2). Fine particle metrics (PM_2.5_, SO_4_^2–^, BC, and OC) were highly correlated with each other but not with PNC, ozone, and temperature. PNC and mean temperature had a high negative correlation (Table 2); ozone and temperature were moderately positively correlated in the non-winter seasons [Spearman’s rho (*r*_S_) = 0.44].

**Table 2:** Summary statistics and Spearman correlation coefficients of daily mean air pollutant concentrations and meteorological variables in Boston, Massachusetts (USA): September 2006 through July 2010.

Summary statistics									*r*_S_										
Exposure	No. of daily environmental measurements	Mean	First quartile	Median	Third quartile	SO_4_^2–^	BC	OC	PNC	Ozone	Temp
PM_2.5_ (μg/m^3^)		1,341		8.6		5.3		7.3		10.5		0.75		0.66		0.61		–0.13		0.09		0.27
SO_4_^2–^ (μg/m^3^)		1,079		2.2		1.0		1.6		2.6		1		0.52		0.51		–0.22		0.04		0.22
BC (μg/m^3^)		1,402		0.60		0.37		0.53		0.76				1		0.60		0.01		–0.21		0.28
OC (μg/m^3^)		935		3.5		2.6		3.3		4.3						1		–0.01		0.12		0.08
PNC (1,000/cm^3^)		1,390		14.5		9.6		14.3		18.5								1		–0.39		–0.77
Ozone (ppb)		1,430		25		18		24		32										1		0.37
Mean temperature (°C)		1,461		11.2		3.8		11.8		19.0												1


**Figure 1:**
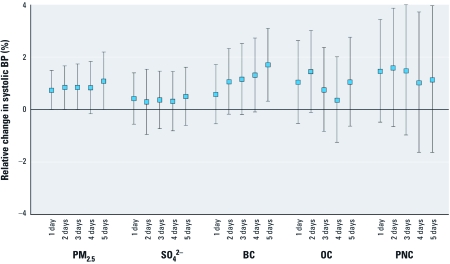
Estimated relative change (and 95% CI) of SBP related to short-term increases of PM, per IQR of the exposure metric. Estimates adjusted for ozone (5-day mean), temperature (4-day mean), season, age, sex, BMI, and years of diabetes.

BP tended to increase with elevations in levels of fine particle mass averaged over the 1–5 days before examination (Figure 1, Table 3). Elevations in PM_2.5_ and BC were most consistently and precisely linked to elevated BPs in the full model. For example, an IQR increase in ambient PM_2.5_ (3.54 μg/m^3^) and BC (0.25 μg/m^3^) in the previous 5 days predicted an increase in SBP of 1.1% (95% CI: 0.0%, 2.2%) and 1.7% (95% CI: 0.3%, 3.1%), respectively (Table 3). This represents absolute increases in SBP of 1.4 mmHg (95% CI, 0.0, 2.9 mmHg) and 2.2 mmHg (95% CI, 0.4, 4.0 mmHg), respectively. Effects on central mean arterial BP derived from the central BP curve showed a similar pattern, but were lower overall.

**Table 3:** Estimated relative change [percent change (95% CI)] of arterial BP related to increases in the 2- and 5-day mean of PM, ozone, and temperature.

Exposure	IQR of exposure metric	SBP	DBP	Central mean BP
PM_2.5_ (μg/m^3^)								
2-day mean		4.2		0.8 (0.0, 1.7)		0.8 (0.1, 1.5)		0.4 (–0.4, 1.1)
5-day mean		3.5		1.1 (0.0, 2.2)		1.0 (0.1, 1.9)		0.6 (–0.4, 1.7)
SO_4_^2–^ (μg/m^3^)								
2-day mean		1.5		0.3 (–1.0, 1.5)		0.6 (–0.4, 1.7)		–0.2 (–1.4, 0.9)
5-day mean		1.2		0.5 (–0.6, 1.6)		0.4 (–0.5, 1.3)		–0.1 (–1.1, 1.0)
BC (μg/m^3^)								
2-day mean		0.34		1.1 (–0.2, 2.3)		0.7 (–0.3, 1.8)		0.7 (–0.5, 1.8)
5-day mean		0.25		1.7 (0.3, 3.1)		1.5 (0.3, 2.6)		0.9 (–0.4, 2.2)
OC (μg/m^3^)								
2-day mean		1.5		1.4 (–0.1, 3.0)		1.2 (–0.1, 2.5)		0.7 (–0.8, 2.3)
5-day mean		1.3		1.1 (–0.6, 2.8)		1.4 (0.0, 2.8)		0.3 (–1.3, 2.0)
PNC (1,000/cm^3^)								
2-day mean		7.3		1.6 (–0.6, 3.9)		0.2 (–1.7, 2.1)		0.8 (–1.3, 2.9)
5-day mean		6.6		1.1 (–1.6, 4.0)		0.1 (–2.2, 2.5)		0.2 (–2.4, 2.9)
Ozone (ppb)								
2-day mean		13.7		–0.6 (–2.5, 1.4)		0.1 (–1.5, 1.7)		–0.3 (–2.1, 1.5)
5-day mean		13.3		–4.0 (–6.6, –1.4)		–2.0 (–4.2, 0.2)		–2.8 (–5.2, –0.3)
Temperature (°C)								
2-day mean		12.3		–1.0 (–3.4, 1.6)		–0.5 (–2.5, 1.6)		–1.1 (–3.4, 1.3)
5-day mean		11.5		–1.8 (–4.5, 1.0)		–1.7 (–3.9, 0.5)		–2.1 (–4.6, 0.4)
All estimates are adjusted for season, age, sex, BMI, and years of diabetes. PM estimates are also adjusted for ozone (5-day mean) and temperature (4-day mean). Ozone and temperature effects are also adjusted for each other and for PM_2.5_ (5-day mean).								

**Figure 2:**
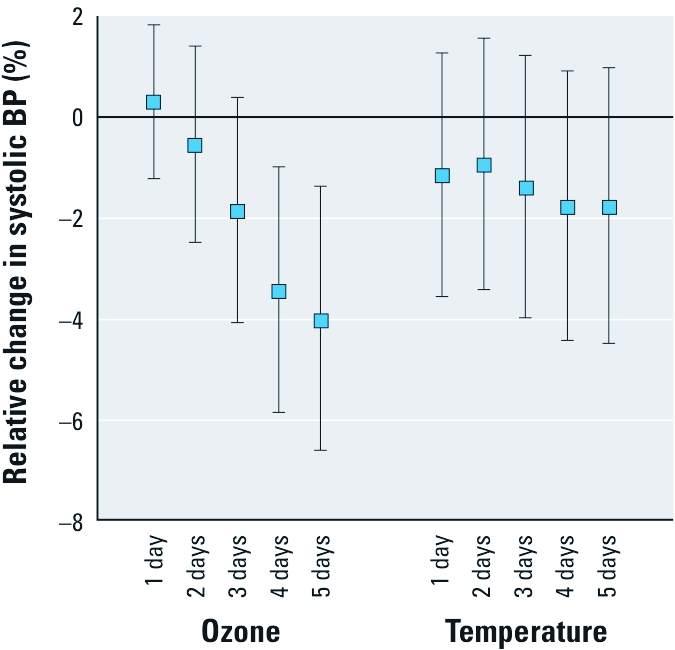
Estimated relative change (and 95% CI) of SBP related to short-term increases of ozone and temperature, per IQR of the exposure metric. Estimates adjusted for each other and for PM_2.5_ (5-day mean), season, age, sex, BMI, and years of diabetes.

In contrast to the rise in BP with increasing PM pollution, increases of mean ozone levels over the 3–5 days before examination were associated with considerable decreases in BP (Table 3, Figure 2). For an IQR increase in the 5-day mean of ozone, SBP, DBP, and central mean arterial BP decreased by 4.0%, 2.0%, and 2.8%, respectively (Table 3), leading to an absolute decrease of 5.2 mmHg (95% CI: –8.6, –1.8 mmHg), 1.5 mmHg (95% CI: –3.1, 0.2 mmHg), and 2.6 mmHg (95% CI: –4.9, 0.3 mmHg), respectively. Higher ambient temperatures during the 1–5 days before the examination were also associated with lower BP. For example, an increase in 5-day mean temperature by 11.5°C (IQR) was associated with relative and absolute decreases in SBP of 2.5% (95% CI: –5.1%, 0.2%) and 3.2 mmHg (95% CI: –6.6, 0.3 mmHg), respectively, in the model without ozone. The BP-lowering effect of temperature was attenuated when simultaneously adjusting for ozone (–1.8%; 95% CI: –4.5%, 1.0%; Table 3, Figure 2).

In subjects with higher baseline BP, BP increased more after PM exposure (Table 4). On the other hand, decreases in BP after high ozone and high temperature exposure were considerably stronger in those with lower baseline BP. Furthermore, subjects with higher HbA1c at baseline tended to react more strongly to increases in PM exposure. Other personal characteristics and the season during which the examination took place did not consistently modify associations (Table 4).

**Table 4:** Effect modification of PM, ozone, and temperature effects on SBP.

PM_2.5_			BC			Ozone			Temperature		
Characteristic	Percent change (95% CI)	*p*-Value	Percent change (95% CI)	*p*-Value	Percent change (95% CI)	*p*-Value	Percent change (95% CI)	*p*-Value
Baseline SBP																
< 132 mmHg		–0.9 (–2.2, 0.3)		—		–0.6 (–2.1, 0.9)		—		–7.0 (–9.6, –4.3)		—		–5.2 (–8.0, –2.4)		—
≥ 132 mmHg		2.9 (1.6, 4.2)		< 0.001		4.5 (2.8, 6.2)		< 0.001		–0.9 (–3.7, 1.9)		< 0.001		3.0 (0.0, 6.1)		< 0.001
Antihypertensive medication																
No		1.1 (–0.4, 2.6)		—		2.3 (0.3, 4.3)		—		–2.9 (–6.1, 0.3)		—		–1.7 (–5.3, 2.0)		—
Yes		1.1 (–0.1, 2.3)		0.991		1.5 (0.1, 3.0)		0.421		–4.4 (–7.0, –1.7)		0.223		–1.8 (–4.6, 1.0)		0.937
AI																
< 26.2%		1.1 (–0.2, 2.4)		—		1.6 (0.0, 3.1)		—		–4.4 (–7.2, –1.6)		—		–2.0 (–4.9, 1.0)		—
≥ 26.2%		1.1 (–0.2, 2.4)		0.988		1.9 (0.2, 3.7)		0.725		–3.4 (–6.3, –0.5)		0.357		–1.5 (–4.6, 1.7)		0.727
BMI																
< 30.6 kg/m^2^		0.9 (–0.5, 2.3)		—		2.0 (0.3, 3.7)		—		–3.6 (–6.5, –0.6)		—		–1.8 (–4.9, 1.4)		—
≥ 30.6 kg/m^2^		1.2 (–0.1, 2.6)		0.686		1.4 (–0.4, 3.1)		0.536		–4.4 (–7.3, –1.5)		0.515		–1.8 (–4.8, 1.4)		0.988
HbA1c																
< 6.7%		0.6 (–0.6, 1.8)		—		0.9 (–0.7, 2.6)		—		–4.3 (–7.1, –1.4)		—		–2.9 (–5.8, 0.1)		—
≥ 6.7%		2.0 (0.5, 3.5)		0.058		2.4 (0.8, 4.0)		0.082		–3.7 (–6.5, –0.9)		0.597		–0.4 (–3.5, 2.8)		0.089
Diabetes mellitus																
< 8 years		1.1 (–0.1, 2.4)		—		1.8 (0.3, 3.4)		—		–3.9 (–6.6, –1.1)		—		–1.7 (–4.5, 1.2)		—
≥ 8 years		1.0 (–0.3, 2.4)		0.839		1.5 (–0.1, 3.2)		0.724		–4.3 (–7.2, –1.3)		0.733		–2.0 (–5.2, 1.3)		0.851
Estimates are given per IQR of the exposure metric. The *p*-value refers to the interaction term. Estimates are displayed for the 5-day mean concentration of PM_2.5_, BC, and ozone and for the 4-day mean of ambient temperature. All estimates are adjusted for season, age, sex, BMI, and years of diabetes. PM estimates are also adjusted for ozone (5-day mean) and temperature (4-day mean). Ozone and temperature effects are also adjusted for each other and for PM_2.5_ (5-day mean).																

Effect estimates were slightly reduced but not qualitatively changed after excluding outliers (data not shown). Different model specifications, such as the inclusion of additional personal characteristics, did not have an impact on the results (data not shown). When we included fixed effects for participants, effect estimates were unchanged compared with the main analysis with the exception of estimates for 5-day average BC and PM_2.5_, which were increased in magnitude with somewhat improved precision. For example, for an interquartile increase in 5-day BC, the linear mixed model predicted a 1.7% (95% CI: 0.3%, 3.1%) increase in SBP, whereas a fixed effect model predicted a 1.9% (95% CI: 0.5%, 3.4%) increase in SBP. Reducing the study area to a smaller buffer of 15 km around the central monitoring site led to a substantial (20%) decrease in sample size (leaving 257 of 322 observations). The magnitude of the effects were generally reduced, but precision decreased as well, making interpretation difficult (data not shown).

## Discussion

To the best of our knowledge, this is the first study to report evidence of independent and diametrically opposed effects of ambient PM pollution, gaseous pollution (ozone), and climate (temperature) on persons with diabetes. Although particle pollution, specifically traffic-related particles, was associated with increased BP, ozone and (less consistently) high temperatures were associated with reduced BP. Estimated effects of particle pollution on BP were stronger in subjects with suboptimal BP and diabetes control.

*Clinical implications.* Persons with diabetes mellitus appear to be particularly vulnerable to environmental stressors such as air pollution and heat, as suggested by an increased risk of cardiovascular hospital admissions and all-cause mortality (Goldberg et al. 2001; O’Donnell et al. 2011; Schifano et al. 2009; Zanobetti et al. 2009). Although the potential biological mechanisms that mediate this hypothesized increased susceptibility remain unclear, it is well known that persons with diabetes suffer from impaired BP regulation with increased risk of both hypertension and orthostatic hypotension. Because of autonomic dysregulation, endothelial dysfunction, fluid shifts, and medication effects, they may be unable to accommodate in a timely or effective manner to environment-related increases or decreases in BP.

The clinical implications of environment-induced BP changes could be substantial. An increase in left ventricular afterload caused by PM-induced elevated BP could lead to an increase in myocardial oxygen demand that might not be adequately compensated in persons with compromised vascular function, which is common with diabetes. Thus, PM pollution-induced increases in left ventricular afterload could partially explain the increased vulnerability of persons with diabetes to cardiovascular death, myocardial infarction, and stroke with short-term increases in air pollution (Goldberg et al. 2001; O’Donnell et al. 2011; Zanobetti et al. 2009). Repeated environmentally induced elevations in BP would also lead to repeated increases in arterial wall stress, potentially causing reactive hypertrophy of arteriolar smooth muscle cells, a process that contributes to the fixation of chronically elevated pressures. Epidemiological evidence for a chronic increase in arterial BP is emerging from population-based studies (Chuang et al. 2011; Fuks et al. 2011). We saw smaller estimated effects on central mean arterial pressure derived from the aortic PP curve. Because central mean arterial pressure is not only a function of SBP but also largely influenced by the diastolic decay of arterial BP, this outcome measure is not sensitive enough to reflect small elevations in peak SBP.

Although long-term BP reduction is generally beneficial to patients with diabetes, acute decreases might be harmful if they lead to symptomatic hypotension or impaired cardiac or cerebral perfusion. Our data suggest that ozone and high temperature lowered BP only when BP was already either optimally controlled (American Diabetes Association 2010) or relatively low. Further lowering of BP in these circumstances could lead to adverse events. Patients with diabetes tend to have depressed heart rate variability and autonomic dysfunction and frequently are already taking beta blockers (Bell 2009). Particularly if their glucose levels are not well controlled, they also may become fluid depleted and hypovolemic during periods of concurrent high temperature and high ozone concentrations. Because their BP compensatory mechanisms are deficient, they may be less able to respond with appropriate vasoconstriction (Pop-Busui 2010; Ribeiro-Oliveira et al. 2008). Even subtle reductions in central mean arterial pressure could contribute to lower diastolic filling of the coronary arteries, with deleterious effects on myocardial oxygen supply, or to reduced cerebrovascular perfusion. Because ozone and PM air pollution are not correlated, contrary effects on BP are not expected to cancel each other out but may act independently, exacerbating the already compromised BP regulation of diabetic patients.

*Biological mechanisms.* We have previously documented elevations in BP with increased PM pollution in patients undergoing cardiac rehabilitation, and now a growing body of literature reflects the replication of our findings in other populations (Brook et al. 2011; Chuang et al. 2010; Delfino et al. 2010; Dvonch et al. 2009; Madsen and Nafstad 2006) and demonstrates mechanisms for BP effects in animal models (Bartoli et al. 2009). In the present study, a PM-induced increase in BP occurred within 24 hr after elevated exposures and was sustained for up to 5 days. Activation of pulmonary reflexes, imbalance in autonomic function, activation of the renin-angiotensin system, and an increase in endothelin, resulting in an increased vasomotor tone, are possible biological mechanisms (Brook 2008; Liu et al. 2009; Luttmann-Gibson et al. 2010). An effect sustained for up to 5 days is more likely to be mediated by slower-acting mechanisms such as the induction of systemic inflammation (Dubowsky et al. 2006). Because poor glucose control is linked to increased systemic inflammation, adverse effects of pollution-induced inflammation and increases in BP could be reduced by improved glucose control. This may explain why study participants with HbA1c < 6.7% had less of an increase in BP in response to increased PM pollution.

Ozone is an oxidant with immediate effects on pulmonary reflexes (Lee and Pisarri 2001), but its effect on BP is less well documented. Ozone may also be a marker for secondary pollution exposure related to regional pollution, including transported traffic emissions. In single-pollutant models, Chuang et al. (2010) showed a small increase in DBP with increasing ozone in a Taiwanese cross-sectional study, and Zanobetti et al. (2004) found an increase in DBP in a panel of cardiac rehabilitation patients. However, a lack of adjustment for other air pollutants might have confounded the ozone estimates in these studies. Other studies did not show an independent effect of ozone, but these studies either were limited to acute effects within hours of exposure (Brook et al. 2009) or might have suffered from substantial exposure misclassification because of a presumably high prevalence of air-conditioning in a study population of elderly subjects with coronary heart disease living in retirement homes in California (Delfino et al. 2010). In a controlled chamber study of normal young adults, we found that combined ozone and concentrated ambient PM exposure increased DBP, but ozone alone did not (Fakhri et al. 2009).

Ozone is a known local vasodilator that has been used in the form of reinfused ozonated plasma in therapy for local peripheral ischemia (Valacchi and Bocci 2000). However, inhalation of ambient ozone is not comparable to these therapeutic measures, and we can only speculate regarding potential biological mechanisms explaining our findings. Ozone selectively activates a subset of bronchopulmonary C-fibers in mice, which can lead to bradycardia, a fall in cardiac output, and bronchial vasodilation that increases airway blood flow despite systemic hypotension (Lee and Pisarri 2001; Taylor-Clark and Undem 2010). Thus, it is physiologically plausible that ozone, independent of the peripheral vasodilation caused by high temperatures (Halonen et al. 2011), may cause systemic hypotension in humans. It is, however, unclear how this relates to the associations we observed after prolonged exposure of several days.

*Limitations and strengths.* Limitations of this study include possible exposure misclassification due to the use of central monitoring data. Several mechanisms of exposure misclassification have to be considered. First, the central site measurement does not reflect the absolute level of personal exposure, which probably differs from the central measurements because of characteristics of the individuals’ homes (e.g., proximity to traffic) and differences in personal behavior (e.g., heating and ventilation habits, leisure time and occupational activities). Therefore, we used a random participant intercept in the analysis to account for differences of the absolute level of exposure of each subject. Second, the study relied upon temporal variation in exposure before each of the five clinic visits, which were scheduled 2 weeks apart. This temporal variation in exposure from week to week is mainly influenced by meteorology on a regional scale. We therefore excluded subjects residing outside a 25-km circle around the monitoring site to ensure homogeneous meteorological conditions within our study area. Finally, differential exposure misclassification may have resulted if participants spent more time indoors on very hot days when ambient ozone concentrations are high, thus reducing their personal exposure relative to ambient levels. Interestingly, the estimated effect of ozone was not modified by season, as would be expected with increasing exposure misclassification on hot summer days.

Other limitations include the comparatively small sample size. The high negative correlation between temperature and PNC limited our power to examine the concurrent effect of both of these environmental exposures.

Strengths of this study include its repeated measures study design, which enables control of potentially confounding personal characteristics in a real-life setting. In contrast to controlled exposure studies, this study estimates the actual effect of ambient air pollutants in a subgroup of the population. We minimized non-air-pollution sources of variance by strict inclusion and exclusion criteria. Further analyses, making use of the comprehensive assessment of exposures and intermediary factors that was conducted in this study, will allow investigations of the mechanisms and biological pathways of PM effects.

## Conclusions

We report evidence that PM air pollution raised arterial BP, whereas ozone decreased BP, including central mean arterial pressure, in persons with T2DM. These estimated effects appeared to be independent of each other and were of a magnitude that could result in clinically significant hemodynamic consequences for this vulnerable population. Optimal BP and glucose control may reduce adverse effects of traffic pollution on increases in BP, but diabetes patients with baseline BP that is optimally controlled or relatively low by American Diabetes Association standards may be at highest risk for further lowering of BP by ozone or high temperature.
